# A Century of Epidemiological Advances in Cutaneous and Visceral Leishmaniasis in Algeria

**DOI:** 10.1155/japr/2102270

**Published:** 2025-06-24

**Authors:** Naouel Eddaikra, Razika Benikhlef, Denis Sereno

**Affiliations:** ^1^Laboratory of Parasitological Eco-Epidemiology and Populations Genetics, Institute Pasteur of Algeria, Algiers, Algeria; ^2^GoInsect: Infectiology and Entomology Research Group, Montpellier, Occitanie, France; ^3^Research Unit Host-Vector-Parasite-Environment Interactions in Trypanosomatidae Neglected Tropical Diseases, INTERTRYP, IRD, CIRAD, University of Montpellier (I-MUSE), Montpellier, France

**Keywords:** Algeria, epidemiology, *Leishmania infantum*, *Leishmania major*, *Leishmania tropica*, leishmaniasis, reservoir, sandfly

## Abstract

Leishmaniasis is a group of diseases transmitted by sandflies, affecting humans and animals, with three clinical presentations: cutaneous, mucosal, and visceral. The disease is caused by the parasite *Leishmania* and is a significant global health issue, with approximately two million cases annually and 350 million people at risk. The disease is endemic in 98 countries, including Algeria, which is a hotspot. In Algeria, three species of *Leishmania* (*Leishmania major*, *Leishmania infantum*, and *Leishmania tropica*) cause cutaneous and visceral leishmaniasis, with the cutaneous one being highly endemic. In Algeria since the 19th century, research on leishmaniasis has been intensive, and the review was aimed at compiling over a century of research to provide updated knowledge on transmission, diagnosis, treatment, and epidemiology in this country.

**Summary**

• This article discusses the epidemiology of cutaneous and visceral leishmaniasis (VL) in Algeria.

• Leishmaniasis is a group of vector-borne diseases affecting humans and animals, presenting in three main clinical forms: cutaneous, mucosal, and visceral.

• The pathogen *Leishmania*, which is carried by sandflies of the genera *Phlebotomus* and *Lutzomyia*, is responsible for the disease.

• Algeria has a high incidence of cutaneous leishmaniasis (CL), and two species of *Leishmania* are responsible for this disease.

• The aim of this review is to provide an updated understanding of the disease components, transmission patterns, diagnosis, treatment, and epidemiology in Algeria.

## 1. Introduction

Leishmaniasis is a group of vector-borne diseases affecting both humans and animals. It comprises three main clinical forms: CL, mucosal leishmaniasis, and VL, reflecting the specific tissue location affected in the host [1, 2]. Among the 53 species of *Leishmania* described, 31 are known to infect mammals, including 23 that are pathogenic to humans [3–5]. *Leishmania* are present in diverse ecosystems and are capable of infecting a wide range of mammals. They are transmitted through various zoonotic and anthroponotic cycles involving 189 domestic and wild mammalian reservoirs belonging to 10 different orders [6, 7]. They are transmitted to mammalian reservoirs by blood-sucking Diptera belonging to the genus *Phlebotomus* in the old world and various genera in the new world, including *Lutzomyia* and *Migonemya* [8]. At least 98 of the more than 800 species of sandflies described are considered potential or proven *Leishmania* vectors worldwide [1, 9, 10]. In humans, disease severity varies from mild to severe. The progression of the disease and its treatment are influenced by factors such as the host's immune status and general health, coinfections with other pathogens, components of sandfly saliva, virulence, and immune-evasive features of the *Leishmania* parasite [11–13].

The disease is a serious public health problem worldwide, yet it remains largely neglected and under-researched. The disease is endemic in 98 countries across the tropics, subtropics, and Mediterranean basin, with an estimated 2 million cases reported annually and approximately 350 million people at risk [14, 15]. In 2020, the World Health Organization (WHO) reported 208,357 new cases of CL and 12,838 new cases of VL. Approximately 73% of new CL cases were recorded in the Eastern Mediterranean region (EMR) and 19% from the African Mediterranean region (AMR). The EMR and Algeria are perceived as ecoepidemiological “hotspots” because of their high prevalence of CL cases. These two regions account for 79% of all CL cases, with a total of 162,371 reported cases. In addition, seven countries—Afghanistan, Algeria, Brazil, Colombia, Iraq, Pakistan, and the Syrian Arab Republic—each reported more than 6000 CL cases, collectively accounting for more than 80% of all CL cases worldwide [16].

In North Africa, leishmaniases cause a scourge in human health with complex epidemiological profiles and disease presentations [17–20]. In Algeria, two clinical presentations have been reported: CL and VL. The CL form is more endemic than the VL form [14, 16]. The first case of leishmaniasis in Algeria was described by Hamel in 1860, followed by the Sergent brothers in 1911 [21]. Since then, the progression of the disease has been variable, particularly for the cutaneous form, with Algeria having one of the highest incidences in the world [16, 20]. Moreover, canine leishmaniasis (CanL) is also endemic to Algeria, with a significant prevalence reported [22–24].

This review examined the evolution and trends of human and animal leishmaniasis in Algeria. Its aim is to provide an updated and comprehensive understanding of the disease components, transmission patterns, diagnosis, treatment, and epidemiology.

## 2. Visceral and Cutaneous Human Leishmaniases

### 2.1. Geographical Situation

Algeria is a vast country with a diverse ecosystems and biotopes. As the largest country in Africa and the Mediterranean Basin, it spans approximately 2.38 million square kilometers. The long Mediterranean coastline is complemented by a southern region that encompasses a significant portion of the Sahara Desert. To the north, the Tell Atlas and Saharan Atlas mountains rise, while to the south, two parallel sets of relief extend eastward, separating vast plains and highlands. The area from the coast to the Tell Atlas is characterized by fertile land, while south of the Tell Atlas lies a steppe landscape that transitions into the Saharan Atlas and ultimately, the Sahara Desert. Algeria's diverse vegetation includes coastal, mountainous, and grassy desert-like regions that together support a rich and varied array of wildlife.

### 2.2. CL in Algeria

**Figure 1:**
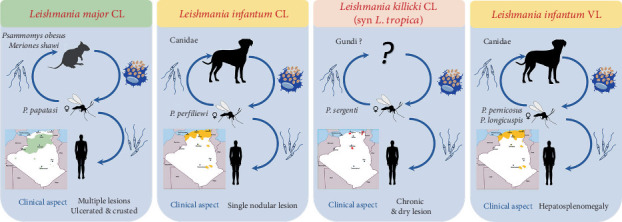
Epidemiological components of the three forms of CL and of VL in Algeria with annotation to different geographical regions.

Three species of *Leishmania* are responsible for CL, each of which presents distinct clinical forms (Figure 1). The most abundant species is *Leishmania major*, a zoonotic cutaneous leishmaniasis (ZCL), endemic in the south, particularly in the Sahara and highland regions [25, 26]. A sporadic form of CL caused by *Leishmania infantum* was found in the northern part of the country [27, 28].

In the southern region of Algeria, particularly in the Ghardaia Department, a chronic form of CL induced by *Leishmania killicki* (also known as *Leishmania tropica*) was discovered in 2005. *L. killicki* is a member of the *L. tropica* complex that commonly coexists with *L. major* in the same area [29, 30]. More recently, molecular detection of *L. killicki* (Syn *L. tropica*) has also been reported in northern regions [31, 32].

CL due to *L. major* is the oldest reported form of leishmaniasis in Maghreb. It was first described in 1860 in Biskra, Algeria, and was known as “Clou de Biskra” [33]. In 1884, Deperet and Boinet referred to the lesion as “Bouton de Gafsa” in Gafsa, Tunisia [17]. This form of leishmaniasis was also identified in southern Morocco in 1914 [34].

#### 2.2.1. Presentation of CL Clinical Forms

*L. major* causes localized cutaneous lesions (LCLs). The typical lesion caused by this parasite is of the “wet” type, characterized by an infiltrated nodule with a large central ulceration covered by a crust. These lesions are commonly characterized by severe inflammation and ulceration and typically resolve within a period of 2–8 months. At the beginning, a small itchy red papule appeared in the exposed area. The lesion extended in surface area and depth, leading to central crusty ulceration. The lesion is typical and is known as the oriental sore [28, 35]. Lesions can differ in terms of their severity, number, clinical presentation (whether they are dry or wet), and the time it takes for them to heal on their own [28]. Often, numerous lesions develop and merge, becoming confluent and susceptible to secondary infection, especially in individuals who lack immunity. Lesions of this nature tend to heal slowly and, in some cases, leave behind significant, disfiguring, or impairing scars. The typical incubation period was less than 4 months [9]. Lesions typically emerge after summer, mainly during September, October, and November, following an incubation period of a few weeks to a few months after infectious bites occur primarily at the end of summer [36]. In addition to this classical ulcerative form, a large but less frequent clinical polymorphism of lesions caused by *L. major* has been observed. These include various localizations on exposed parts of the body, with lesions presenting as erythemato-squamous, papulous, eczematoid, or recidivans [37, 38]. A complicated form of CL has recently been reported in patients with diabetes, leading to leg amputation [35].

*L. infantum* is the most frequent species responsible for sporadic cutaneous leishmaniasis (CLS) in northern Algeria [28, 39]. The first identification of *L. infantum* was made by Belazzoug in 1985 using multilocus enzyme electrophoresis (MLEE) [40]. Lesions typically present as single nodules with minimal inflammation, and four clinical cases have been described: papular, ulcerated, erythematous-scaly, and infiltrated [41].

*L. tropica*: The first molecular detection of *L. tropica* in Algeria was performed in 2008 in the Constantine Department in the northeast of the country. In 2009, the parasite was isolated and identified by MLEE in the Ghardaia Department, located in the southern gateway, and identified as *L. tropica* (Syn *L. killicki*) Zymodeme MON-281. This species coexists sympatrically with *L. major* [29, 31]. Over the past decade, *L. tropica* has been identified in other departments in the north (Tipaza) and northeast (Annaba) [32, 42, 43]. Lesions caused by *L. tropica* exhibit a broad clinical spectrum, but the chronic dry type is dominant, usually healing spontaneously within a year or for an extended period, often resulting in disfiguring scars. These lesions are characterized by ulcerocrusted nodules that are relatively dry and preferentially located on the face [44]. The incubation period typically ranges from 2 to 8 months [9, 17]. In addition, clinical polymorphisms are observed in Tunisia, Morocco, and Algeria, with erythematous, papulonodular, nodular, and mucosal lesions [45].

#### 2.2.2. Epidemiology of CL in Algeria

**Figure 2:**
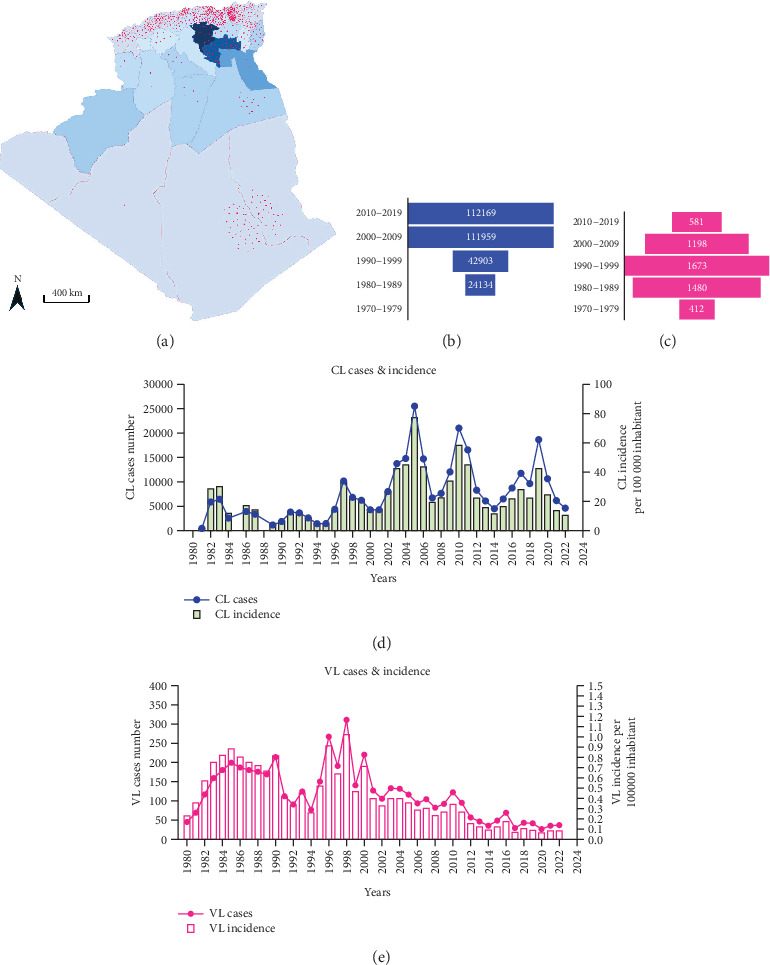
Evolutionary aspects of incidence and cases number of CL (blue) and VL (pink) during five decades (1970–2022). (a) Cumulative number of cases of CL in blue and VL in red over 50 years. (b) Cumulative number of cases of CL per decade. (c) Cumulative number of cases of VL per decade. (d) Evolution of incidence and number of cases of CL. (e) Evolution of the incidence and number of cases of VL.

Algeria is one of the most endemic countries for CL worldwide [16]. It is a notifiable disease, and the incidence of CL is much higher than that of VL. All departments are affected, with a higher incidence in the highlands and departments of Batna, Biskra, and Msila [20]. A rather worrying extension toward the north (the village of El Mhir) was reported in 2012 [46]. The number of cases reported by the health authorities has increased by 80% over the past four decades (Figure 2b). The incidence per 100,000 inhabitants increased from 28.59 in 1982 to 24.23 in 2020, with peaks in 1983, 1986, 1997, 2005, 2010, and 2019 (Figure 2d). After almost 40 years, more than a quarter of a million cases have been recorded in the country. The highest number of cases was reported in 2005, with over 25,000 cases and an incidence rate of 76.68 per 100,000 inhabitants (Figure 2d) [20, 38]. The epidemic explosion of CL in Algeria and the Maghreb is probably multifactorial and mainly follows agricultural development in arid zones favorable to vectors and reservoirs, but uncontrolled urbanization has introduced humans into the wild biotopes of the species in question [17, 38].

### 2.3. VL in Algeria

Only *L. infantum* is responsible for VL in Algeria, and the first case was reported by Lemaire in 1911, in a child from Kabylie [47, 48]. In 1984, Belazzoug confirmed the role of *L. infantum* Nicolle, 1908 as the causative agent of human VL in Algeria using MLEE of 12 enzymes [49, 50]. He also demonstrated a dog-to-human parasite transfer by finding the same species in dogs, thereby irrefutably confirming the reservoir role of this animal [40]. VL is prevalent throughout the northern part of Algeria. This distribution fits with the humid and subhumid bioclimatic stages, but many cases have been reported in semiarid and arid regions, such as the wilayas of Msila, Batna, and Biskra, which are primarily known for the presence of ZCL caused by *L. major* [39]. Between 2010 and 2021, the disease has spread to other departments where it occurs sympatrically with CL, including Tébessa, Jijel, Oum El Bouaghi, Relizane, Sétif, and Mila [20, 51–58] (Figure 2a). Additionally, cases continue to be reported in the Hoggar and Tassili N'Ajjar regions in the far south of Algeria.

#### 2.3.1. VL Clinical Form Description

VL, commonly referred to as kala-azar, is a potentially life-threatening condition that can be fatal if left untreated in more than 95% of cases. The disease is characterized by intermittent fevers, significant weight loss, the enlargement of the spleen and liver, and severe anemia [27, 59].

Infection preferentially affects children under 5 years of age and manifests as a symptomatic triad: irregular fever, cutaneous and mucosal pallor, and splenomegaly. Additional symptoms include hepatomegaly, peripheral lymphadenopathy, progressive weight loss, and hemorrhagic signs, with the latter indicating poor prognosis. Hepatomegaly, which is firm, smooth, and painless, is usually moderate, although its volume may exceed that of the spleen in some cases. Significant hepatosplenomegaly results in a bulky abdomen that contrasts with weight loss of the limbs, justifying the term “spider child.” Lymphadenopathy is a rare, firm, mobile, painless, and noninflammatory disease, with variable locations such as cervical, inguinal, or axillary. These lymph nodes do not suppurate or fistulize [60, 61].

In adults, the clinical presentation is less typical than in children. Adults may present with prolonged febrile forms; splenomegaly may be absent or present as the only clinical sign, which is similar to lymphadenopathy. Skin signs can predominate and appear suddenly, complicating diagnosis [60–62].

#### 2.3.2. Epidemiology of VL in Algeria

VL cases have been systematically monitored in Algeria since the early 20th century. Between 1911 and 1933, 18 cases of VL were reported [60, 63], and from 1965 to 1974, 497 cases were diagnosed [60, 64]. Several epidemiological surveys on VL were conducted between 1972 and 1990 in major Algerian hospitals [62, 65]. In addition, the National Institute of Health Statistics (INSP) reported the number of cases and incidence rates from 1980 to 2020. These reports indicate an expansion from historical foci in Kabylie (Tizi Ouzou and Bejaia) to central regions (Blida, Chlef, Medea, and Tipaza) and the northeastern part of northern Algeria, with scattered cases in the western regions, including Oran and Tlemcen [23].

During the last decade (2010–2019), central departments were more affected than northern ones, reflecting a change in the geographical distribution pattern. The highest incidence was recorded in the Tell area between 1984 and 2020, with a maximum of 310 cases reported in 1998 (Figure 2c). However, the number of VL cases has significantly decreased since then. From 2010 to 2020, there were 606 reported cases compared to the1673 cases reported between 1990 and 1999. The incidence rate has also dropped significantly, from 1.02 per 100,000 inhabitants in 1998 to 0.05 per 100,000 inhabitants in 2020 (Figure 2e). A reduction in the occurrence of the disease has been noticed in the historical hotspots of Bouira and Tizi Ouzou. The incidence has decreased from 1.55 to 0.79 cases per 100,000 inhabitants during the 1994–2003 period and from 1.43 to 0.28 cases during the 2004–2013 period. From 2010 to 2021, most cases were found in foci known to have high CL incidence, such as Setif, Mila, and Biskra [20, 51–58] (Figure 2a). In addition, 11.3% of VL cases were reported in Hoggar and Tassili N'Ajjar, in the far south of Algeria.

## 3. *Leishmania* Diagnosis and Species Identification

The first and most critical diagnostic step for clinicians is to consider the diagnosis of CL when assessing a chronic skin lesion in a person with potential exposure in an endemic region [16]. As CL is endemic across all departments in Algeria, most diagnostic laboratories microscopically confirm CL by identifying amastigotes in scrapings stained with Giemsa. Few laboratories use materials from the ulcer base in combination with microscopy for culture, which increases diagnostic sensitivity and allows for the isolation of strains for more specific studies, including species identification by PCR-RFLP and MLEE. Some specialized laboratories use parasite cultivation for the diagnosis, species identification, and epidemiological studies of leishmaniasis in Algeria. Parasites were isolated from CL lesions or through hemoculture and bone marrow aspiration for VL and were cultivated on NNN (Novy–MacNeal–Nicolle) with coagulated rabbit serum medium. Detection of parasite DNA from lesions using PCR is generally more sensitive for diagnosing CL [66, 67]. However, culture and PCR testing are technically challenging, expensive, and currently not practical for all diagnostic laboratories in Algeria.

VL requires biopsy of the liver, spleen, and bone marrow. In general, the suspicion of VL is primarily based on clinical symptoms such as fever, fatigue, loss of appetite, and enlargement of the liver and spleen. Microscopic examination of the bone marrow smears is typically performed. The duration of the incubation period typically ranges from 3 to 7 months [68]. Primary biological indicators include an inflammatory condition with a markedly elevated erythrocyte sedimentation rate, hyperproteinemia, polyclonal hypergammaglobulinemia, and tricytopenia (anemia, leukopenia, and thrombocytopenia). Ninety percent of pediatric cases exhibit tricytopenia. Diagnosis requires the identification of parasites from Giemsa-stained bone marrow smears. Serological tests have also been used to detect blood antibodies [28, 69]. Some diagnostic tests such as the indirect fluorescent antibody test (IFAT), ELISA, and Western blotting (WB) have demonstrated high accuracy but are dependent on equipment that is not ideally suited for field use [9].

In Algerian VL patients, especially those who are immunosuppressed, a positive serological result leads to a strong diagnostic assumption. IFAT on cultured promastigote forms remains the standard approach; however, ELISA assays, whose specificity and sensitivity vary with the antigens, are gradually replacing it [61, 70]. The highly sensitive WB technique is highly specific and sensitive, enabling the differentiation between sick and asymptomatic carriers. However, this confirmatory test is reserved for specialized laboratories.

At the National Reference Center (Pasteur Institute of Algeria), MLEE and PCR-RFLP were performed for species-level identification. MLEE, based on isoenzyme analysis, requires parasite culture. It is a comprehensive method for *Leishmania* typing, particularly the MON system developed in Montpellier, France, which uses 15 enzymes. Protein extracts were analyzed by electrophoresis, and the migration distance of each enzyme band from the origin (anode) was determined. The set of bands defines the zymodeme. Species attribution is made by comparing the obtained profiles with reference *Leishmania* strains [71, 72]. The second method uses restriction fragment length polymorphism (RFLP) that often targets include the 18S ribosomal RNA gene, the 39-nucleotide small mini exon gene, or the ITS1 region, between the 18S ribosomal RNA and 5.8S ribosomal RNA genes. These regions are highly repetitive and conserved, with sequence variability allowing for species-specific identification [68, 73, 74]. The PCR-RFLP developed by Schönian et al. [75] is the most widely used. In Algeria, PCR-RFLP is based on the amplification of the ITS1 region before restriction with the Hae III enzyme. This method efficiently discriminates between the three Algerian endemic species: *L. infantum*, *L. major*, and *L. tropica*.  Table 1 summarizes the findings and references regarding *Leishmania* in Algeria, based on clinical forms, humans, reservoirs, and vectors.

Despite all these techniques, the diagnosis could be improved in the field laboratory by using techniques that do not require a thermocycler, such as loop-mediated isothermal amplification (LAMP). Furthermore, monitoring the treatment response would be greatly improved by the introduction of qPCR from blood samples.

## 4. Treatment and Chemotherapeutic Failure/Drug Resistance in Algeria

Controlling and treating leishmaniasis primarily relies on the use of affordable medications, as effective vaccines are currently unavailable. Current chemotherapy options include medications that have been employed since the 1950s, such as pentavalent antimony (Sb(V)) compounds, including Pentostam and Glucantime, pentamidine, different compositions of the antifungal amphotericin B, and miltefosine [92]. In North Africa, the standard treatment for both the cutaneous and visceral forms of leishmaniasis involves the use of pentavalent antimonial compounds. In Algeria, healthcare services provide treatment at no cost to patients. According to the Algerian National Essential Drug List, both meglumine antimoniate and conventional amphotericin B are listed.

The Ministry of Health's protocol for treating all forms of CL involves the administration of Glucantime (Sb(V)) at a dose of 20 mg/kg/day via intramuscular injection for 15 consecutive days. This regimen is recommended for cases with multiple lesions or lesions located on the face. For single lesions, the Health Ministry recommend intradermal (intralesional) administration of 1.5–2 mL of Glucantime twice per week for 4 weeks. Additionally, hydrogen peroxide (H_2_O_2_ 10 vol) or cryotherapy may be used. For VL, the Ministry recommends intramuscular injections of Glucantime (20 mg Sb(V)/kg/day) for 28 days in accordance with the WHO's protocol. In cases of nonresponsiveness, intravenous infusion of Fungizone (amphotericin B) at a dose of 1 mg/kg/day for 15 days is recommended [20].

A lack of responsiveness to Sb(V) during the treatment of patients with CL caused by *L. major* (ZCL) is well documented in Algeria, particularly in the M'sila region. According to a study carried out in 1986 on 97 children administered 60 mg/kg/day of meglumine antimoniate for 15 days, no significant difference was observed between the treatment and the placebo groups [89]. In vitro experiments on intracellular amastigotes revealed that all strains of *L. major* isolated from these children showed low sensitivity to Sb(V)-containing drugs, including Glucantime [89]. More recently, it has been observed that 9% of children with VL do not respond to antimonial treatment [62]. A survey of antimonial susceptibility in *Leishmania* isolates covering a 30-year period disclosed an increasing frequency of CL *Leishmania* isolates expressing a decreased susceptibility toward antimony-containing drugs [20].

These observations highlight the ongoing challenges in the treatment of leishmaniasis and underscore the need for continued research and the development of more effective therapeutic strategies. Many Algerian research teams are screening *Leishmania* with plant extracts or synthetic molecules to identify molecules with therapeutic potential [93, 94].

## 5. *Leishmania* Reservoir and Hosts in Algeria

### 5.1. Dogs and Wild Canidae

CanL is a severe disease that affects several million domestic dogs and can be fatal if left untreated [95]. The dog is the primary reservoir of VL, caused by *L. infantum* in the “Old World” and *Leishmania chagasi* (Syn *L. infantum*) in the “New World” [96]. In Mediterranean countries, infection rates can reach up to 60%, including at least 2.5 million seropositive dogs [24, 97]. In the early 20th century, dogs were identified as reservoirs of CanL in Tunisia (1908) and Algeria (1910) [47, 98]. In addition, the presence of *L. infantum* has been well-documented in wild animals such as jackals [83].

#### 5.1.1. Epidemiology and Transmission

CanL is endemic in Algeria. *L. infantum* is responsible for CanL [50, 82, 99]. Five zymodemes have been isolated from dogs in Algeria: MON-24 [87], MON-34, MON-77 [39], MON-80 [76], and MON-281 [86]. The enzymatic polymorphism is relatively high in Algeria and Spain compared to other Mediterranean countries, with Algeria having the highest polymorphic index (PI = 0.06) in the region, higher than Spain's (PI = 0.03) [22]. The transmission of *L. infantum* occurs through the bites of female *Phlebotomus perniciosus* and *Phlebotomus longicuspis* [39, 78].

#### 5.1.2. Clinical Manifestations

The incubation period of CanL ranged from a few months to several years. Clinical manifestations vary and include cutaneous, mucocutaneous, and visceral forms. Common clinical signs include the following:

• Onychogryphosis (abnormal development and curving of claws)
• Poor general condition
• Swollen lymph nodes
• Weight loss
• Cutaneous lesions such as ulcers, dermatitis, periorbital scaling, or alopecia [23, 24, 100, 101]

Dog's infection can manifest as subclinical infection, a self-limiting illness, or a serious and life-threatening disease [95, 96]. Some dogs are unable to mount an effective cell-mediated immune response and instead exhibit a powerful but ineffective humoral response, leading to significant clinical symptoms. Conversely, dogs may remain infected for years or even their entire lives without showing any clinical symptoms or lesions [102].

#### 5.1.3. Diagnosis

The prompt diagnosis of infected dogs is essential. Serology is the most commonly used diagnostic method in Algeria. However, specialized laboratories also perform the following methods:

• IFAT
• Microscopic examination of lymph node and bone marrow smears
• Polymerase chain reaction (PCR) [23, 103]

#### 5.1.4. Prevalence

The first reported prevalence of CanL was 11.7%, documented in the Kabylie region of northern Algeria and was associated with a VL incidence of 2.6 cases per 100,000 inhabitants [63, 82]. Investigations performed between 1910 and 2020 revealed fluctuating and disparate prevalence rates, ranging from 2.52% to 74.44% (Table 2). Studies on asymptomatic dogs have shown positive rates of 12.04% and 18% [23, 110], respectively.

In the Algiers region, Aït-Oudhia et al. [22] reported that 58.8% of dogs with positive serology results were asymptomatic, whereas only 15.4% exhibited more than three clinical signs. Most studies indicate a high prevalence of asymptomatic dogs, whereas 78% of the studied dog population presents symptoms (Table 3). Stray dogs, which accounted for 73% of the mixed breeds, showed the highest prevalence (11.7%). This is likely due to their roaming behavior, which increases their exposure to infected sandfly bites, and their poor physical condition, which makes them more susceptible to infection [111, 112].

### 5.2. Other Animal Reservoirs

Leishmaniasis is primarily a zoonotic disease, meaning that it is transmitted between animals and humans. Mammals other than humans serve as the primary reservoir hosts, with humans acting as incidental hosts. In Algeria, rodents are the main reservoirs for ZCL caused by *L. major*, and their population dynamics are closely linked to the distribution of the disease. Two rodent species, *Psammomys obesus* and *Meriones shawi*, are the principal reservoirs identified in Algeria [80, 113]. Understanding the ecology and behavior of these rodent species is crucial for predicting and managing ZCL outbreaks.

*P. obesus* (fat sand rat) is found in the semidesert regions of northern Sahara, where halophilic Chenopodiaceae, which constitute its primary food source, are present. Despite their presence in salt pans, these rats exhibit limited social behavior, primarily restricted to predator warnings, and each rat inhabits a separate burrow. Breeding activities occur mainly during winter, resulting in a high prevalence of infection among juvenile rats. Infected animals are typically near the end of their 18-month lifespan [6]. Populations of *P. obesus* are notably unstable, with significant fluctuations in density and local extinctions. Despite these population dynamics, it remains the primary reservoir host for *L. major*, responsible for disease outbreaks in other countries such as Saudi Arabia, Libya, Tunisia, and Algeria. In 2009, studies of reservoir hosts revealed the expansion of *L. major* ZCL to northern Algeria, as evidenced by the discovery of *P. obesus* in the El M'hir area. This is the first report of this species in the northern part of the active focus in Msila [46].

Gerbils (*Meriones* sp.) may play a temporary role as reservoirs of *Leishmania*, such as *Meriones hurrianae*, which is implicated in a *L. major* outbreak in northwest India [6]. In North Africa, *M. shawi* is the primary animal host involved in two significant epidemics: in Tata, southern Morocco [114], and in M'sila, Algeria [113]. In M'sila and the neighboring northern region of Setif, epidemic outbreaks are primarily associated with the proliferation of *M. shawi* during rainy years [46, 115]. The proliferation of this rodent species is driven by irrigation, agricultural development, uncontrolled urbanization, and urban waste accumulation [46, 116].

In Msila, *L. major* high infection rates have been reported for *P. obesus* (29.6%) and *M. shawi* (27%) [46, 115]. These exceptionally high infection rates confirm the primary reservoir role that supports the high endemicity of ZCL in this region.

## 6. Research on Sandfly as Vectors of *Leishmania* in Algeria

*Leishmania* vectors are mostly prevalent in warm regions of Asia, Africa, Australia, southern Europe, and the Americas. The common name “sandfly” is derived from the yellow sandy color of this small vector. Sandflies belong to the order Diptera, the suborder Nematocera, the family Psychodidae, and the subfamily Phlebotominae [117]. The first evidence of sandflies as vectors of leishmaniasis in Algeria can be traced back to the early 20th century, when an experimental lesion in a volunteer was induced using seven specimens of *P. papatasi* obtained from Biskra [91]. Later, Parrot et al. documented the infection of four *P. perniciosus* females out of 53 that fed on a CanL dog in Algiers [118]. In 1931, 58 females of *P. perniciosus* were used in an experiment where they were fed infected dogs. As a result, 14 of the females spontaneously contracted infections by *L. infantum* promastigotes [119]. This was the initial confirmation of *L. infantum* presence in Algeria. *P. longicuspis* was suggested to be a possible vector of VL, alongside *P. perniciosus* following the observation of a natural infection rate of 16.5% in *P. longicuspis* females fed dogs with leishmaniasis [77].

In Kabylia, *L. infantum* MON-1 was isolated from *P. perniciosus*, confirming its role as a vector for VL in Algeria [78]. In 1992, *L. major* was successfully isolated from *P. papatasi* in Biskra, providing empirical evidence supporting Sergent's earlier observations that *P. papatasi* is the primary vector in this region [91]. Additionally, dermotropic *L. infantum* was successfully isolated from *Phlebotomus perfiliewi* in Tenes [85]. *L. infantum* DNA was further detected in *P. longicuspis* from endemic VL foci in Kabylia [120].

Using molecular tools, the evidence of the zoonotic cycle of *L. killicki* (Syn *L. tropica*) was provided in Ghardaia, Algeria, with *P. sergenti* acting as a vector and gundi rodents as reservoirs [30].

**Figure 3:**
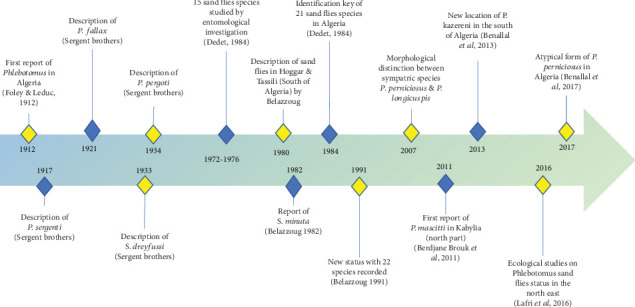
Timeline of sandfly studies describing a century of the major discoveries in Algeria from 1912 to 2023.

From 15 sandfly species reported to be endemic in Algeria in 1972, the checklist was further updated to 21 species in 1984, and an identification key for Algerian sandflies was set up (Figure 3) [121]. In 1991, the checklist was further amended for 22 reported species [122]. In 2011, *Phlebotomus mascittii* was first reported in Algeria in an endemic VL focus of Kabylia [123], and *Phlebotomus kazeruni* was identified in Tamanrasset, increasing the phlebotomine fauna to 24 species [124]. In addition, the presence of an atypical form of *P. perniciosus* has been reported in Algeria [125]. Recently, the accuracy of MALDI-TOF MS for identifying field-caught sandflies has been proven [126].

## 7. Prevention and Control

Preventing and controlling leishmaniasis requires a multifaceted approach that encompasses prompt diagnosis and effective treatment, vector control, disease surveillance, management of animal reservoirs, and social mobilization. Although efficacious and safe pharmacological treatments are available, their implementation poses significant challenges. Vector control strategies, such as insecticide spraying and the deployment of insecticide-treated bed nets, are essential for reducing sandfly populations. Robust disease surveillance systems are crucial for monitoring incidence and responding promptly to outbreaks. Furthermore, the management of animal reservoirs requires strategies that are specifically adapted to local ecological and epidemiological contexts. Social mobilization and partnerships with a wide range of stakeholders, including community education initiatives and collaboration with other disease control programs, are imperative for the success of leishmaniasis control efforts.

### 7.1. Reservoir Control

Zoonotic leishmaniasis depends on the specific reservoir involved. For VL, in which dogs serve as significant reservoirs, euthanasia of infected dogs is the primary control method. In contrast, control measures for CL have focused on rodents, with strategies tailored to the biology of each species. For instance, controlling *P. obesus* involves the destruction of its burrows and the elimination of Chenopodiaceae plants. In 2003, a pilot project in the five most severely affected cities of M'Sila engaged 396 unemployed young individuals in a public works program to remove Chenopodiaceae before the transmission season, covering over 3600 ha. This effort resulted in a reduction in cases from 1391 in 2003 to 965 in 2004, representing a 31% decrease [116]. Control methods for *M. shawi* include flooding burrows, which, although detrimental to agriculture, effectively reduce the rodent populations.

### 7.2. Vector Control

Vector control aims to prevent the spread of leishmaniasis by targeting sandflies, primarily at the domestic level. These measures include insecticide spraying, insecticide-treated bed nets, environmental management, and personal protective equipment. According to the WHO (2018), phlebotomine sandflies are highly sensitive to insecticides, although some resistance to DDT has been reported [127, 128]. Insecticides can be applied to the internal walls of houses (indoor residual spraying (IRS)) or impregnated into bed nets, curtains, bedsheets, and clothing. IRS is the most commonly used intervention for controlling endophilic sandflies; however, it requires frequent application, which reduces its long-term sustainability [129, 130].

In response to the CL outbreak in 2005, which affected the entire country but more significantly the departments of Msila and Biskra with over 25,000 cases, the Algerian government initiated a nationwide campaign to combat leishmaniasis in 12 wilayas across the highlands, steppe, and south. This large-scale deltamethrin spraying campaign was conducted in collaboration with six government departments: agriculture, health, interior, defense, environment, and housing. Additionally, the Ministry of Health established a comprehensive national action plan to tackle leishmaniasis, which included intensive antivector control campaigns and extensive awareness-raising efforts through various communication channels such as radio broadcasts, posters in healthcare facilities, and television programs.

Consequently, the incidence of CL decreased from 79 cases per 100,000 inhabitants in 2005 to 53 cases per 100,000 inhabitants in 2006 [46]. Chemical treatment vector control measures covered approximately 22,800 outbreaks out of the 335,699 planned nationally, achieving a coverage rate of 6.8%. This rate is continuously increasing, with the goal of reaching 80% coverage by the end of the campaign.

## 8. Conclusion

Leishmaniasis remains a critical public health challenge in Algeria, marked by a troubling increase in the incidence and geographical spread of CL, even in cases of VL decline. The persistence of this neglected disease is exacerbated by environmental changes, urbanization, and lack of effective antileishmanial treatments. Therefore, there is an urgent need for enhanced epidemiological research, robust surveillance, and comprehensive control systems. Advances in molecular techniques now facilitate precise identification of *Leishmania* species, paving the way for more effective and rational therapeutic management.

Current CL treatment guidelines are inadequate and rely on poorly designed trials. It is imperative to conduct large, standardized trials to assess promising treatments, focusing on less toxic drugs and painless modalities in children. Strengthened vector control measures, vaccine development, and improved diagnostics are essential for reducing the incidence and morbidity of CL. Individual prophylaxis is crucial, primarily through the use of insecticide-treated mosquito nets, indoor spraying with approved insecticides, and maintenance of environmental hygiene. This includes garbage incineration, wastewater drainage, and restoration of dilapidated houses, as recommended by the WHO.

Immediate and concerted efforts in these areas are vital for curbing the growing threat of leishmaniasis in Algeria. Enhanced epidemiological research and robust surveillance systems will provide necessary data for implementing effective control strategies.

## Figures and Tables

**Table 1:** Overview of findings performed in Algeria, on *Leishmania* clinical forms, species, reservoirs, vectors, and methodologies for parasite identification.

**Clinical form**	**Localization**	**Species/zymodeme**	**Reservoir/parasite species-zymodeme**	**Vectors species/parasite**	**Methods of identification**	**Reference**
Visceral leishmaniasis (VL)	Intracellular in white blood cells, liver, spleen, and lymph nodes	*L. infantum* *MON-1* *MON-24* *MON-80*	Dogs: *Canis familiaris* (*L. infantum*) (MON-1 and MON-24) Golden jackal *Canis aureus* (*L. infantum* MON-1)	*Phlebotomus perniciosus* *L. infantum MON-1* *Phlebotomus longicuspis* *L. infantum* MON-1	MLEE	[39, 49, 60, 76–78]

Cutaneous leishmaniasis of the north (CLN)	Intracellular in the skin	*L. infantum* *MON-1* *MON-24* *MON-80* *MON-33* *MON-34* *MON-77*	Dog *Canis familiaris* (MON-1& MON-24) Golden jackal *Canis aureus* (*L. infantum* MON-1)	*Phlebotomus perfilewi* *L. infantum MON-24*	MLEE	[39, 76, 79–85]

Canine leishmaniasis	Intracellular in white blood cells, liver, spleen, and lymph nodes	*L. infantum* *MON-1* *MON-24* *MON-80* *MON-33* *MON-34* *MON-77* *MON-281*	Dog *Canis familiaris* *L. infantum* MON-1	*Phlebotomus perfilewi* *Phlebotomus perniciosus* *Phlebotomus longicuspis*	MLEE	[22, 39, 59, 63, 76–78, 82–84, 86, 87]

Zoonotic cutaneous leishmaniasis	Intracellular in the skin	*L. major* *MON-25* *MON-269*	Rodents: *Psammomys obesus* (*L. major MON-25*) *Meriones shawi* (*L. major* MON-25)	*Phlebotomus papatasi* (*L. major MON-25*)	MLEE	[39, 71, 80, 88–91]

Cutaneous leishmaniasis due to *L. tropica*	Intracellular in the skin	*L. killicki* (Syn *L. tropica*) *MON-301* *MON-306*	*Ctenodactylus gundi?* *L. killicki* (Syn *L. tropica*) MON-301?	*Phlebotomus sergenti*	MLEE ITS1 PCR-RFLP Real-time Topoisomerase II gene PCR and sequencing	[29, 30, 32, 42]

**Table 2:** Canine leishmaniasis studies in Algeria from 1910 to 2022.

**Period**	**Region**	**Asymptomatic**	**Positive/total (%)**	**Diagnosis**	**References**
1910–1913	Algiers	—	25/833 (3%)	Direct exam and culture	[98]
1945–1950	Algiers	—	35/444 (7.8%)	Direct exam and culture	[104, 105]
1972–1973	Algiers	—	9/357 (2.5%)	Direct exam and culture	[106]
1975–1984	Tizi Ouzou	—	37.5%	37.5% IFAT	[82]
1990–1997	Algiers	167 (25%)	666/1800 (37%)	05 lymph node culture 666 (37%) IFAT	[107]
October 2004 to June 2005	Algiers	100%	56/462 (12.04%)	56 (12.04%) IFAT	[108]
November 2005 to June 2008	Algiers	267 (58.8%)	454/1810 (25.1%)	50 (2.76%) lymph node culture 332 (18.3%) IFAT 407 (22.5%) ELISA	[86]
2007–2010	Tizi Ouzou	—	60 (9.95%)	60/603 (9.95%) IFAT	[109]
2015–2018	Tizi Ouzou	92%	55 (74.44%)	170 (28.23%) IFAT 55 (74.44%) PCR	[103]
2019	Bouira	100%	17/94 (18%)	9 (10%) IFAT 17 (18%) ELISA 3 (3%) PCR	[110]
2018	Bouira, Tizi Ouzou, and Setif	115 (23.3%)	81/227 (36%)	81/227 (36%) IFAT 32/227 (14%) PCR	[101]
February 2018 to December 2020	Tiaret	75 (46.58%)	93 (68.32%)	55% (26 sur 47) lymph node cytology 10 (7%) IFAT 14 (10%) ELISA 93 (68%) PCR	[100]

**Table 3:** Canine leishmaniasis symptoms according to four main studies in Algeria.

**Symptoms**	**Harrat and Belkaid [** 105 **]**	**Ait Oudhia et al. [** 86 **]**	**Mouloua et al. [** 102 **]**	**Bia et al. [** 99 **]**
Cachexia	446 (67%)	30%	28 (5.01%)	23 (18%)
Skin signs	346 (52.1%)	22.7%	67 (11.99%)	26 (20.31%)
Hair loss	315 (47.2%)			21 (16.40%)
Onychogryphosis	293 (44%)	11.7%	34 (6.08%)	81 (63.28%)
Lymphadenopathy	290 (43.6%)	15.1%	15 (2.68%)	39 (30.46%)
Epistaxis	153 (23%)		12 (2.15%)	
Eye signs	67 (10%)	4.6%	6 (1.06%)	8 (6.25%)
Asymptomatic	167 (25%)	267 (58.8%)	439 (78.53%)	75 (46.58%)

## Data Availability

The data that support the findings of this study are available on request from the corresponding author. The data are not publicly available due to privacy or ethical restrictions.
